# Noncoding RNAs in Cardiac Hypertrophy and Heart Failure

**DOI:** 10.3390/cells11050777

**Published:** 2022-02-23

**Authors:** Peilei Lu, Fan Ding, Yang Kevin Xiang, Liying Hao, Meimi Zhao

**Affiliations:** 1 Department of Pharmaceutical Toxicology, School of Pharmacy, China Medical University, Shenyang 110122, China; lupeilei95@hotmail.com (P.L.); fding@cmu.edu.cn (F.D.); 2 Department of Pharmacology, University of California, Davis, CA 95616, USA

**Keywords:** heart failure, cardiac hypertrophy, noncoding RNAs

## Abstract

Heart failure is a major global health concern. Noncoding RNAs (ncRNAs) are involved in physiological processes and in the pathogenesis of various diseases, including heart failure. ncRNAs have emerged as critical components of transcriptional regulatory pathways that govern cardiac development, stress response, signaling, and remodeling in cardiac pathology. Recently, studies of ncRNAs in cardiovascular disease have achieved significant development. Here, we discuss the roles of ncRNAs, including microRNAs (miRNAs), long noncoding RNAs (lncRNAs), and circular RNAs (circRNAs) that modulate the cardiac hypertrophy and heart failure.

## 1. Introduction

Cardiac hypertrophy is an essential milestone of many heart diseases, including hypertension [1], myocardial infarction (MI) [2], and aortic stenosis [3,4]. It may be the initial adaptive response to maintaining cardiac function; sustained hypertrophy is often accompanied by maladaptive cardiac remodeling that ultimately leads to heart failure and sudden death [5,6]. Many effective cardiovascular drugs, such as angiotensin-converting enzyme (ACE) inhibitors, angiotensin receptor blockers (ARBs), β-blockers, mineralocorticoid receptor antagonists, and sodium-glucose co-transporter-2 (SGLT2) inhibition, are available for patients with heart failure with reduced ejection fraction (HFrEF) [7,8,9]. However, few evidence-based therapeutical plans are available for heart failure with preserved ejection fraction (HFpEF), acute heart failure, or the preventive management of individuals with cardiovascular risk factors [10,11]. Thus, delaying or preventing heart failure is increasingly important in patients at risk, and should be prioritized in future research studies. One way to achieve this goal is to block or reverse pathological cardiac hypertrophy in heart failure.

At the cellular level, cardiac hypertrophy is featured by increased cardiac myocyte size, sarcomere assembly, and fetal cardiac gene re-expression. Pathological stimuli induce these alterations by activating intracellular signaling pathways and transcriptional mediators in cardiac myocytes [6,12]. Hence, cardiac hypertrophy is associated with changes in gene expression.

In recent years, the development of next-generation sequencing technologies has led to an explosion of newly identified noncoding RNAs (ncRNAs), such as microRNAs (miRNAs), linear long noncoding RNAs (lncRNAs), and circular noncoding RNAs (circRNAs). Unlike messenger RNAs (mRNAs), ncRNAs do not encode proteins, but rather act as epigenetic regulators [13], post-transcriptional modifiers [14], and translational coordinators of gene expression [15,16]. Mounting evidence highlights the aberrant expression of ncRNAs in cardiac development and cardiac diseases using RNA-sequencing technologies and genomewide profiling approaches. For example, the expression of 43 out of 428 miRNAs, including miR-1, -19, -133, and -108, is altered in human heart disease [17]. Ounzain et al. revealed hundreds of novel heart-specific lncRNAs with potential roles in pathological remodeling in a mouse myocardial infarction (MI) model [18]. Tan et al. provided a detailed circRNA expression landscape in human hearts [19]. Dong et al. established a functional paradigm for identifying novel circRNAs in human dilated cardiomyopathy [20]. ncRNAs may provide therapeutic targets and serve as biomarkers in diagnosis and prognosis [21,22,23]. The development of RNA interference (RNAi) drugs, which use the recently discovered endogenous short interfering RNA pathway, suggests that therapy with ncRNAs is a new frontier of disease treatment [24]. However, the role and function of ncRNAs in cardiac hypertrophy and heart failure remain to be illustrated. Here, we review the potential roles of these three classes of well-described ncRNAs as potential targets for therapy. 

## 2. Biogenesis and Function of ncRNAs

On the basis of the length of nucleotides, ncRNAs are categorized into small (<200 bp) and long (>200 bp) RNAs. Small RNAs, comprising 19–25 bp miRNAs, target RNA stability and translation, while long RNAs are composed of linear long noncoding (lncRNAs) and circular (circRNAs) RNAs [16,25].

### 2.1. MiRNAs

**Figure 1:**
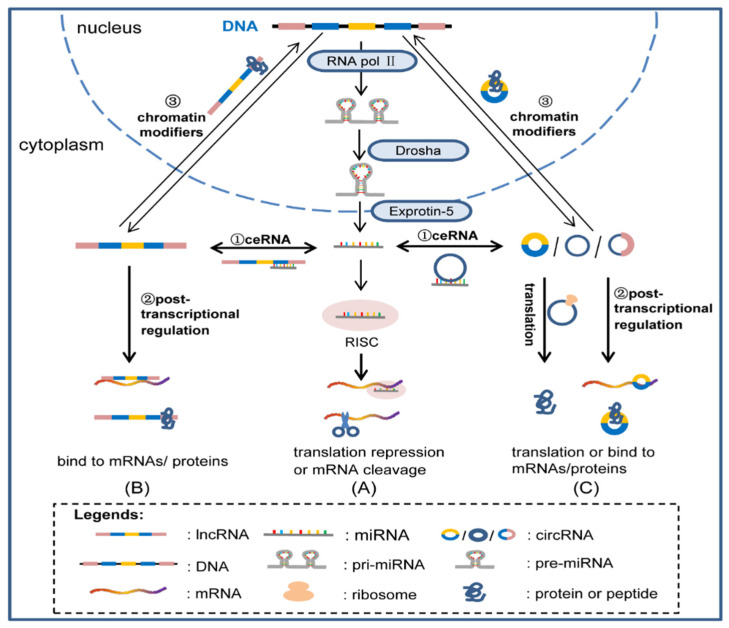
Biogenesis and function of ncRNAs. ncRNA genes are transcribed by RNA Pol Ⅱ to generate pri-RNAs. Drosha crops pri-RNAs into pre-RNAs, and pre-RNAs are exported from nucleus to cytoplasm by Exportin-5. (**A**) miRNAs direct RISC to downregulate gene expression by mRNA cleavage or translation repression. (**B**) LncRNAs act as signals, and decoy proteins and other RNAs to regulate translation. LncRNAs can bring together multiple proteins to affect histone modifications. (**C**) CircRNAs act as miRNA sponges and interact with RNA binding proteins, and function as protein scaffolds and modifiers of parental gene expression. CircRNAs can also be translated into proteins.

MiRNAs are endogenous, single-stranded, small noncoding RNA molecules that suppress gene expression at the post-transcriptional level by targeting specific mRNAs [26]. RNA polymerase II transcribes miRNA genes to generate primary transcripts (pri-miRNAs). Drosha crops pri-miRNAs into precursor miRNAs (pre-miRNAs), which are exported from the nucleus to the cytoplasm by Exportin-5. Lastly, pre-miRNAs are cleaved by the RNase III enzyme dicer to produce mature miRNAs. miRNAs can direct the RNA-induced silencing complex (RISC) to downregulate gene expression by post-transcriptional mechanisms: mRNA cleavage or translational repression (Figure 1A) [27,28].

### 2.2. LncRNAs

Most lncRNA species are transcribed by polymerase II. Like mRNAs, many lncRNAs display 5′-end CpG islands and 3′-end poly(A) tails. Unlike mRNAs, they are inefficiently processed and retained in the nucleus, whereas others are spliced and exported to the cytoplasm [29,30]. LncRNAs are important players in a wide range of biological processes by targeting proteins and other RNAs to regulate splicing, translation, and mRNA decay. Moreover, lncRNAs can bind multiple proteins to regulate chromatin modification, genetic imprinting, and cell cycle control. LncRNAs are divided into four archetypes on the basis of their molecular functions: (1) as signals, lncRNA expression can reflect the combinatorial actions of transcription factors or signaling pathways to regulate gene regulation by space and time; (2) as decoys, lncRNAs can titrate transcription factors and other proteins from chromatin or titrate protein factors into nuclear subdomains; (3) as guides, lncRNAs may recruit chromatin-modifying enzymes to target gene promoters in either the Cis or Trans of target genes in the distance; (4) as scaffolds, lncRNAs may nucleate multiple proteins to affect histone modifications [31,32] (Figure 1B).

### 2.3. CircRNAs

CircRNAs range from hundreds to a few kilobases of nucleotides in length. They are generally produced from exon regions and introns, exon–introns, and tRNA intron regions of protein-coding genes. They circularize the 3′ and 5′ ends of the RNAs [33,34]. CircRNAs are generated from exons or introns through multiple mechanisms, and most circRNAs are stable and conserved across different species. CircRNAs can act as miRNA sponges and inhibit the activity of one or multiple miRNAs. For example, circRNA ciRS-7 harbors more than 70 conventional miR-7-binding sites, and it was identified as a miR-7 inhibitor [35,36]. CircRNAs can also interact with RNA-binding proteins and function as regulators of splicing and transcription; for instance, circMbl is strongly and specifically bound by MBL proteins [37] and serves as protein scaffold or modifier during parental gene expression [38,39]. Most circRNAs do not appear to be involved in gene expression, but a few, mainly intron circRNAs, may regulate the expression of host genes, such as ci-ankrd52 [40]. Some circRNAs can also be translated into proteins to regulate gene expression (Figure 1C) [41,42,43]. Yang et al. found that circ-fbxw7 can encode a 21-kDa protein (fbxw7-185aa) through an internal ribosome entry site (IRES) [44]. In addition, compared with miRNAs and lncRNAs, circRNAs are more stable and have longer half-lives, making them abundant in extracellular fluid and easy to detect, indicating that circRNAs can be a better choice for biomarkers of cardiovascular diseases [45,46,47].

## 3. Roles and Mechanism of ncRNAs in Cardiac Hypertrophy and Heart Failure

Cardiac hypertrophy is an early milestone before degeneration to heart failure and a significant risk factor for subsequent cardiac morbidity and mortality. Pathologic cardiac hypertrophy is controlled at three levels: extracellular hypertrophic stimulus signal, cytoplasmic signal transduction, and nuclear gene transcription and post-transcription [48]. 

### 3.1. MiRNAs

**Figure 2:**
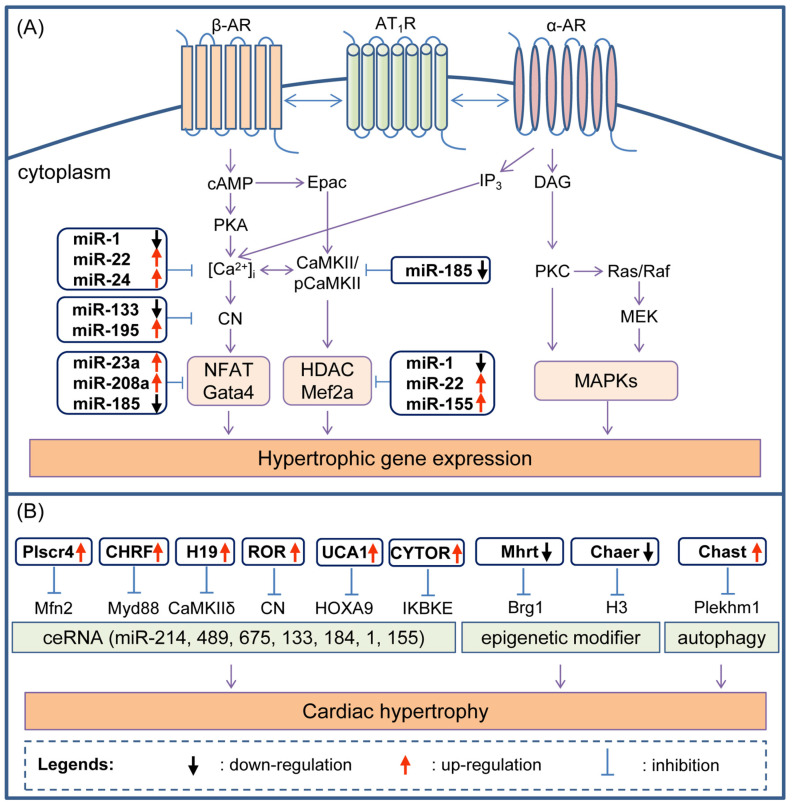
Interaction of partial ncRNAs in cardiac hypertrophy and heart failure. (**A**) MiRNAs regulate hypertrophic Ca^2+^ signaling pathway; (**B**) lncRNAs exert splicing regulation and translational regulation through competing endogenous RNA (ceRNA) mechanism. β-AR, β-adrenoceptors; AT1R, angiotensin Ⅱ type-1 receptor; α-AR, alpha-adrenergic receptor; cAMP, cyclic adenosine monophosphate; PKA, activates protein kinase A; [Ca^2+^]i, intracellular Ca^2+^ concentration; CN, calcineurin; NFAT, nuclear factor of activated T cells; Gata4, GATA-binding factor 4; Epac, exchange factor directly activated by cAMP; CaMKⅡ, calmodulin dependent kinase Ⅱ; pCaMKⅡ, the phosphorylation of CaMKⅡ; HDAC, histone deacetylase; Mef2a, myocyte-specific enhancer factor 2A; IP3, inositol 1, 4, 5-trisphosphate; DAG, dystrophin-associated glycoprotein; PKC, protein kinase C; Ras, Ras family of small GTPases; Raf, Raf proto-oncogene serine/threonine-protein kinase; MEK, mitogen-activated extracellular signal-regulated kinase; MAPKs, mitogen-activated protein kinases; Mfn2, mitofusin-2; Myd88, myeloid differentiation primary response gene 88; CaMKⅡδ, calmodulin dependent kinase Ⅱδ; HOXA9, homeobox A9; IKBKE, I-kappa-B kinase epsilon; H3, histone H3; Plekhm1, Pleckstrin homology domain-containing protein family M member 1.

MiRNAs modulate gene expression at the post-transcriptional level via the activation of intracellular signaling, such as the hypertrophic Ca^2+^ signaling pathway (Figure 2A). Catecholamine stimulates β-adrenoceptors (β-ARs) to activate Gs proteins and generate the second messenger cyclic adenosine monophosphate (cAMP). cAMP binds to and activates protein kinase A (PKA) to phosphorylate Ca^2+^ signaling proteins, such as L-type calcium channel (LTCC), ryanodine receptor (RyR), and sarcoplasmic reticulum Ca^2+^-ATPase (SERCA), thereby increasing intracellular Ca^2+^ concentration ([Ca^2+^]i). Intracellular Ca^2+^, in turn, activates Ca^2+^-dependent calcineurin (CN) and calmodulin (CaM)-dependent kinase II (CaMKII). CN and CaMKII activate transcription factors such as nuclear factor of activated T cells (NFAT) and histone deacetylase (HDAC), respectively, to induce the expression of genes involved in hypertrophy [49,50]. 

Multiple miRNAs are involved in regulating hypertrophy-related pathways. The over-expression of miR-1 attenuates cardiomyocyte hypertrophy in cultured neonatal rat cardiomyocytes and the intact adult heart by downregulating the expression of CaM, GATA-binding factor 4 (Gata4), Mef2, and the CN-NFAT and CaMKII-HDAC transcriptional pathways [51]. Increased miR-22 impairs Ca^2+^ loading into the sarcoplasmic reticulum (SR) and suppresses peroxisome proliferator-activated receptor (PPAR), estrogen-related receptor (ERR), sirtuin 1 (SIRT1), and HDAC [52,53]. MiR-24, a junctophilin-2 (JP2) suppressor, is upregulated in hypertrophy and failing cardiomyocytes [54]. MiR-24 suppression stabilizes JP2 expression and protects the ultrastructure of T-tubule and SR junctions from Ca^2+^ disruption [55]. The downregulation of miR-133 leads to the enhancement of transcriptional repression of CN-NFAT [56]. MiR-195 is upregulated during cardiac hypertrophy, leading to pathological cardiac growth and heart failure in transgenic mice through activated calcineurin A (CnA) [57]. The upregulation of MiR-23a targets NFATc3 directly to promote cardiac hypertrophy [58]. The downregulation of miR-208a is required for the proper expression of cardiac transcription factors Gata4, homeodomain-only protein (Hop), and gap junction protein connexin40 (Cx40) [59]. MiR-185, which is downregulated in hypertrophic hearts, plays an antihypertrophic role by directly targeting CaMKIIδ, NFATc3, and Na^+^-Ca^2+^ exchanger gene (NCX1) [60]. The loss of miR-155 protects the heart from pathological cardiac hypertrophy and failure by targeting the suppressor of cytokine signaling 1 (Socs1) [61,62]. MiRNAs are also involved in signaling pathways other than the hypertrophic Ca^2+^ signaling pathway. Transforming growth factor β1 (TGF-β1)-regulated miR-27b targets PPAR-γ in cardiac hypertrophy [63]. MiR-214 provokes cardiac hypertrophy by repressing the enhancer of homolog 2 (EZH2) [64]. MiR-206 mediates cardiac hypertrophy by inhibiting Forkhead box protein P1 (FoxP1) [65]. MiR-223 directly targets cardiac troponin I-interacting kinase TNNI3K and downregulates cardiac troponin I (cTnI) phosphorylation to suppress cardiomyocyte hypertrophy [66]. Chronic kidney disease can result in left ventricular hypertrophy, in which the expression of miR-30 is downregulated. The knockdown of miRNA-30 in cardiomyocytes leads to hypertrophy by upregulating calcineurin signaling [67].

One of the characteristics of miRNA regulation is that more than one miRNA can affect a single mRNA; for example, many of the above miRNAs were involved in the hypertrophic Ca^2+^ signaling pathway. The other characteristic of miRNA regulation is that one miRNA can influence several targets, such as miR-21. MiR-21 is highly expressed in all main types of cardiovascular cells, and the biological functions of miR-21 were investigated well in cardiovascular disease [68,69]. Depending on cell-specific expression, miR-21 can either protect against hypertrophy and apoptosis [57,70,71] or promote cardiac fibrosis and cardiac hypertrophy in fibroblasts [72]. Danish Sayed et al. identified that miR-21 mediates cardiomyocyte outgrowth by targeting gene SPRY2 [73]. However, miR-21 is upregulated in fibroblasts but not cardiomyocytes in the pressure-overloaded heart model [72]. Silencing of miR-21 in fibroblasts reduces ERK-MAP kinase activity by inhibiting sprouty homolog 1 (Spry1), preventing interstitial fibrosis, and attenuating cardiac dysfunction [72].

### 3.2. LncRNAs

The role of lncRNAs in myocardial hypertrophy has also been extensively studied. As shown in Figure 2B, some lncRNAs can exert splicing and translational regulation through competing endogenous RNA (ceRNA) mechanisms in hypertrophic responses. Jiang et al. detected 16,044 lncRNAs, and established a lncRNA profile of the heart and 29 other tissue types [74]. Approximately 14.7% (2353) of lncRNAs can only be detected in the heart, called heart-specific (HS) lncRNAs. In addition, 1.7% (273) of lncRNAs are expressed in the heart at least five times more than they are in all other tissue types, and 30.1% (4828) of lncRNAs are expressed in the heart at least five times more than the average levels in all other tissue types, which are considered to be heart-enriched (HER) lncRNAs and heart-enhanced (HEH) lncRNAs, respectively. The total number of HS, HER, and HEH is 7454 (46.5%), named heart-elevated (HE) lncRNAs.

LncRNAs can modulate gene expression via several mechanisms, including signaling induced by transcription factors, sponging miRNAs, recruiting chromatin-modifying enzymes, and modifying genomic components [75,76]. Moreover, more than 100 uncharacterized short open reading frames were detected in lncRNA genes, which suggest lncRNAs may be translated into potential micropeptides [77,78].

LncRNA Plscr4 acts as an endogenous sponge of miR-214, the suppresser of mitofusin2 (Mfn2), to maintain mitochondrial homeostasis and exert an antihypertrophic effect in Ang II-treated cardiomyocytes and TAC-induced cardiac hypertrophy [79]. Similarly, lncRNA cardiac hypertrophy related factor (CHRF) acts as an endogenous sponge of miR-489, which regulates the expression of myeloid differentiation primary response gene 88 (Myd88) to facilitate hypertrophy [80]. LncRNA H19 regulates functionality on miR-675 to increase CaMKIIδ expression at both the mRNA and the protein level, leading to enhanced cardiomyocyte hypertrophy [81,82]. The expression of lncRNA ROR negatively correlates with miR-133, enhancing the hypertrophic Ca^2+^ signaling pathway after phenylephrine treatment [83]. The LncRNA of paternally expressed imprinted gene 10 (PEG10) is upregulated in mice with cardiac hypertrophy. PEG10 can regulate the expression of homeobox A9 (HOXA9) to aggravate cardiac hypertrophy; the silence of HOXA9 reverses the cardiac hypertrophy in cardiomyocytes by over-expressing PEG10 [84]. The lncRNA UCA1 can promote the progression of cardiac hypertrophy as an endogenous sponge of miR-184 to enhance the expression of HOXA9 [85]. The expression of miR-1 is negatively correlated with the expression of UCA1 [86]. The lncRNA cytoskeleton regulator RNA (CYTOR), serving as a miR-155 sponge to counteract miR-155-mediated repression of I-kappa-B kinase epsilon (IKKBKE), plays a protective role in I kappa-B kinase (IKKi) and nuclear factor kappa-B (NF-κB) signaling pathway [87].

Some lncRNAs are involved in hypertrophic processes through chromatin remodeling mechanisms. The lncRNA Mhrt antagonizes the function of Brg1, a chromatin-remodeling factor, which is activated by stress to trigger aberrant gene expression, and cardiac hypertrophy and failure [88]. The Chaer lncRNA directly interacts with polycomb repressor complex 2 (PRC2) catalytic subunit through a 66-mer motif and subsequently inhibits histone H3 lysine 27 methylation for epigenetic reprogramming at hypertrophic genes [89]. LncRNAs can regulate cell autophagy. The lncRNA Chast negatively regulates the expression of the autophagy regulator Pleckstrin homology domain-containing protein family M member 1 (Plekhm1) to drive hypertrophy [90]. The long noncoding myosin heavy chain associated RNA transcript Mhrt779 is markedly upregulated under TAC surgery. However, Mhrt779 displays a minimal increase associated with the lower expression of the Nppa and Myh7 genes in the exercise hypertrophy preconditioning group. Silencing of Mhrt779 attenuates the antihypertrophic effect, and overexpression enhances the antihypertrophic effect. Mhry779 can bind Brg1 to inhibit the activation of the Hdac2/Akt/GSK3β pathway induced by pressure overload, acting as an anti-hypertrophic effect [91].

### 3.3. CircRNAs

In 2016, the expression profile of circRNAs in adult mouse hearts was plotted [92]. Jacobi et al. compiled a catalog of 575 candidate circRNAs, and many of these candidates coincided with disease-associated gene loci. For instance, a significant number of candidates originate from the Ryr2, Hectd1, and Ppp2r3a gene loci that are linked to cardiovascular diseases. Wu et al. performed deep RNA-sequencing on ribosomal-depleted RNA isolated from 12 human hearts, 25 mouse hearts, and across a 28-day differentiation time-course of human embryonic stem cell-derived cardiomyocytes. Top highly expressed circRNAs are related to cardiac genes, including Titin (TTN), RYR2, and DMD. The most abundant cardiac-expressed circRNA is a cytoplasmic localized single-exon circSLC8A1-1 [19]. Werfel et al. performed RNA-Seq analysis of ribosome-depleted libraries from rats (neonatal and adult), mice (sham or TAC), and humans (failing, nonfailing). They observed dozens of circRNAs arising from the TTN gene in all three species, which is known to undergo highly complex alternative splicing during heart maturation. They observed extensive differential regulation of TTN circRNAs between neonatal and adult rat hearts, suggesting that circRNA formation could be involved in the regulation of titin splicing [93]. Meng et al. reported that circRNAs are differentially expressed in cardiac hypertrophic cells cultured in the presence of high and normal levels of D-glucose. Five circRNAs, namely, ciRNA261, ciRNA26, circRNA1191, circRNA4251, and circRNA6913, are significantly different. These circRNAs have more than 60 targeted miRNAs, suggesting that they may play a role in myocardial hypertrophy and serve as biomarkers [94]. In 2020, circRNA microarrays on plasma samples obtained from three patients with HF and three healthy controls were investigated. HF patients display 477 upregulated circRNAs, and 219 downregulated circRNAs compared with healthy controls [95]. Yang et al. showed that 401 out of 3323 circRNAs were dysregulated in left ventricular specimens collected from 8-week-old mice with isoproterenol hydrochloride-induced cardiac hypertrophy compared with the controls. Of these, 303 circRNAs were upregulated, and 98 were downregulated [96].

Until now, few studies have fully explained the mechanisms of circRNA in myocardial hypertrophy, especially circRNA related to the Ca^2+^ signaling pathway. MicroRNA-133a (miR-133a) is well-recognized in cardiac hypertrophy [97,98]. Lim et al. found that miR-133a was highly enriched in the fraction of circSlc8a1 pull-down [99]. Next, they show that knockdown of circSlc8a1 attenuates pressure-overload induced cardiac hypertrophy, whereas overexpression of circSlc8a1 results in heart failure. Further research shows circSlc8a1 functions through miR-133a and its downstream targets, including serum response factor (Srf), connective tissue growth factor (Ctgf), adrenoceptor beta 1 (Adrb1), and adenylate cyclase 6 (Adcy6). Meanwhile, circNcx1 (a circRNA transcribed from the sodium/calcium exchanger 1 (ncx1) gene, which is also called solute carrier family 8 member A1 (slc8a1) gene), was able to regulate cardiomyocyte apoptosis by targeting miR-133a-3p-CDIP1 in 2018 [100], but whether it can regulate myocardial hypertrophy by targeting miR-133a is still unknown. circ-HIPK3 affects the concentration of Ca^2+^ in the cytoplasm by the miR-17-3p/ADCY6 axis [101], and the level of circ-HIPK3 in the heart was increased by adrenaline via transcription factor cAMP-responsive element-binding protein 1 (CREB1). The downregulation of circ-HIPK3 can alleviate fibrosis and maintain cardiac function post-MI in mice. Deng et al., therefore, concluded that the increased circ-HIPK3 acts as a helper for adrenaline, but is harmful to the heart in the long run, which may be an ideal therapeutic target of HF. Xu et al. found that circHIPK3 regulates pressure overload-induced cardiac hypertrophy by sponging miR-185-3p and modulating CaSR in neonatal mouse cardiomyocytes [102]. Mouse homolog circ-SIRT1 (mmu_circ_0002354) was downregulated in Ang II-treated H9c2 cells and TAC induced mice model. circ-SIRT1 deficiency was conducive to CH formation, and the overexpression circ-SIRT1 elevates SIRT1 expression by competitively binding with miR-3681-3p/miR-5195-3p or recruits USP22 to stabilize SIRT1 protein in hiPSCCMs [103]. Moreover, many circRNAs can affect ejection fraction, such as heart-related circRNA (HRCR) and circRNA_000203 [104,105], and ejection fraction is closely related to myocardial contractility, which can be affected by Ca^2+^. Therefore, we suspect that these circRNAs may reduce the ejection fraction by regulating the Ca^2+^ signaling pathway. This conjecture needs experimental validation.

## 4. Discussion

The expression of ncRNAs is significantly altered in various cardiac diseases and is an adaptive regulation of the body. Research on ncRNAs in heart failure has rapidly expanded from the initial discovery of the abnormal expression of miRNAs to the role of the newer types of ncRNAs: lncRNAs and circRNAs. More results indicate that ncRNAs play an essential function in heart failure [106,107,108], and the interaction of circRNA/lncRNA/miRNA may provide new ideas for the treatment of heart failure [109]. It is conceivable that modulating ncRNAs may provide potential therapeutic values. First, dysregulated ncRNAs expression could be used as biomarkers to diagnose different kinds of cardiac diseases. Second, ncRNAs could also be potential targets for disease therapies. RNA-targeted therapies provide new ideas for drug discovery involving chemically modified oligonucleotides and novel target-binding motifs [110]. RNA-based drugs have conceptual and practical advantages compared to conventional chemistry-based drugs or antibodies. However, the regulation of ncRNAs is not well-characterized. Additional hurdles also need to be addressed before clinical application, such as off-target effects and potential toxicity [111].

Earlier, ncRNAs were thought of as useless RNAs. ncRNAs act as essential regulators that mediate their functions through complex mechanisms. Recent studies have also indicated that some ncRNAs could be translated into small peptides to regulate gene expression. The roles of peptides encoded by lncRNAs or circRNAs were primarily verified on cancers [112,113,114], but not in cardiac hypertrophy. This review summarized the current understanding of ncRNAs biogenesis and global mechanisms in cardiac hypertrophy and heart failure. The incorporation of ncRNAs within cardiac gene regulatory networks represents a novel venue for therapeutic intervention in clinical heart failure.

## Data Availability

Not applicable.

## References

[1] Grabowski K., Herlan L., Witten A., Qadri F., Eisenreich A., Lindner D., Schädlich M., Schulz A., Subrova J., Mhatre K.N. (2021). Cpxm2 as a novel candidate for cardiac hypertrophy and failure in hypertension. Hypertens. Res..

[2] Zurek M., Johansson E., Palmer M., Albery T., Johansson K., Rydén-Markinhutha K., Wang Q.-D. (2020). Neuregulin-1 Induces Cardiac Hypertrophy and Impairs Cardiac Performance in Post–Myocardial Infarction Rats. Circulation.

[3] Yang Y., Ahn J., Kang D., Ko E., Kim S., Kim T.O., Kim J.H., Lee J., Lee S., Kim D. (2022). Implication of Different ECG Left Ventricular Hypertrophy in Patients Undergoing Transcatheter Aortic Valve Replacement. J. Am. Heart Assoc..

[4] Shimizu I., Minamino T. (2016). Physiological and pathological cardiac hypertrophy. J. Mol. Cell. Cardiol..

[5] Tang X., Wang P., Zhang R., Watanabe I., Chang E., Vinayachandran V., Nayak L., Lapping S., Liao S., Madera A. (2022). KLF2 regulates neutrophil activation and thrombosis in cardiac hypertrophy and heart failure progression. J. Clin. Investig..

[6] Nakamura M., Sadoshima J. (2018). Mechanisms of physiological and pathological cardiac hypertrophy. Nat. Rev. Cardiol..

[7] Nabeebaccus A., Zheng S., Shah A.M. (2016). Heart failure—Potential new targets for therapy. Br. Med. Bull..

[8] Vaduganathan M., Claggett B.L., Jhund P.S., Cunningham J.W., Ferreira J.P., Zannad F., Packer M., Fonarow G.C., McMurray J.J.V., Solomon S.D. (2020). Estimating lifetime benefits of comprehensive disease-modifying pharmacological therapies in patients with heart failure with reduced ejection fraction: A comparative analysis of three randomised controlled trials. Lancet.

[9] Zannad F., Ferreira J.P., Pocock S.J., Anker S.D., Butler J., Filippatos G., Brueckmann M., Ofstad A.P., Pfarr E., Jamal W. (2020). SGLT2 inhibitors in patients with heart failure with reduced ejection fraction: A meta-analysis of the EMPEROR-Reduced and DAPA-HF trials. Lancet.

[10] Rossignol P., Hernandez A.F., Solomon S.D., Zannad F. (2019). Heart failure drug treatment. Lancet.

[11] Mishra S., Kass D.A. (2021). Cellular and molecular pathobiology of heart failure with preserved ejection fraction. Nat. Rev. Cardiol..

[12] Luo Y., Jiang N., May H.I., Luo X., Ferdous A., Schiattarella G.G., Chen G., Li Q., Li C., Rothermel B.A. (2021). Cooperative Binding of ETS2 and NFAT Link Erk1/2 and Calcineurin Signaling in the Pathogenesis of Cardiac Hypertrophy. Circulation.

[13] Pajares M., Alemany-Cosme E., Goñi S., Bandres E., Palanca-Ballester C., Sandoval J. (2021). Epigenetic Regulation of microRNAs in Cancer: Shortening the Distance from Bench to Bedside. Int. J. Mol. Sci..

[14] Chen R.-X., Chen X., Xia L.-P., Zhang J.-X., Pan Z.-Z., Ma X.-D., Han K., Chen J.-W., Judde J.-G., Deas O. (2019). N6-methyladenosine modification of circNSUN2 facilitates cytoplasmic export and stabilizes HMGA2 to promote colorectal liver metastasis. Nat. Commun..

[15] Ponting C.P., Oliver P.L., Reik W. (2009). Evolution and Functions of Long Noncoding RNAs. Cell.

[16] Gomes C., Schroen B., Kuster G.M., Robinson E., Ford K., Squire I.B., Heymans S., Martelli F., Emanueli C., Devaux Y. (2020). Regulatory RNAs in Heart Failure. Circulation.

[17] Ikeda S., Kong S.W., Lu J., Bisping E., Zhang H., Allen P.D., Golub T.R., Pieske B., Pu W.T. (2007). Altered microRNA expression in human heart disease. Physiol. Genom..

[18] Ounzain S., Micheletti R., Beckmann T., Schroen B., Alexanian M., Pezzuto I., Crippa S., Nemir M., Sarre A., Johnson R. (2015). Genome-wide profiling of the cardiac transcriptome after myocardial infarction identifies novel heart-specific long non-coding RNAs. Eur. Heart J..

[19] Tan L.W., Lim B.T., Anene-Nzelu C.G., Ackers-Johnson M., Dashi A., See K., Tiang Z., Lee D.P., Chua W.W., Luu T.D. (2016). A landscape of circular RNA expression in the human heart. Cardiovasc. Res..

[20] Dong K., He X., Su H., Fulton D.J.R., Zhou J. (2020). Genomic analysis of circular RNAs in heart. BMC Med. Genom..

[21] Omura J., Habbout K., Shimauchi T., Wu W.-H., Breuils-Bonnet S., Tremblay E., Martineau S., Nadeau V., Gagnon K., Mazoyer F. (2020). Identification of Long Noncoding RNA H19 as a New Biomarker and Therapeutic Target in Right Ventricular Failure in Pulmonary Arterial Hypertension. Circulation.

[22] Gozdowska R., Makowska A., Gąsecka A., Chabior A., Marchel M. (2022). Circulating microRNA in Heart Failure—Practical Guidebook to Clinical Application. Cardiol. Rev..

[23] Sun C., Ni M., Song B., Cao L. (2020). Circulating Circular RNAs: Novel Biomarkers for Heart Failure. Front. Pharmacol..

[24] Hueso M., Mallén A., Suñé-Pou M., Aran J.M., Suñé-Negre J.M., Navarro E. (2021). ncRNAs in Therapeutics: Challenges and Limitations in Nucleic Acid-Based Drug Delivery. Int. J. Mol. Sci..

[25] Liang J., Li X., Zhang L. (2016). Unraveling the Expression Profiles of Long Noncoding RNAs in Rat Cardiac Hypertrophy and Functions of lncRNA BC088254 in Cardiac Hypertrophy Induced by Transverse Aortic Constriction. Cardiology.

[26] Ottaviani L., Martins P.A.D.C. (2017). Non-coding RNAs in cardiac hypertrophy. J. Physiol..

[27] Liao C., Gui Y., Guo Y., Xu D. (2016). The regulatory function of microRNA-1 in arrhythmias. Mol. BioSyst..

[28] Bartel D.P. (2004). MicroRNAs: Genomics, Biogenesis, Mechanism, and Function. Cell.

[29] Qin T., Li J., Zhang K.-Q. (2020). Structure, Regulation, and Function of Linear and Circular Long Non-Coding RNAs. Front. Genet..

[30] Statello L., Guo C.-J., Chen L.-L., Huarte M. (2021). Gene regulation by long non-coding RNAs and its biological functions. Nat. Rev. Mol. Cell Biol..

[31] Thum T., Condorelli G. (2015). Long Noncoding RNAs and MicroRNAs in Cardiovascular Pathophysiology. Circ. Res..

[32] Wang K.C., Chang H.Y. (2011). Molecular Mechanisms of Long Noncoding RNAs. Mol. Cell.

[33] Zhao G. (2018). Significance of non-coding circular RNAs and micro RNAs in the pathogenesis of cardiovascular diseases. J. Med. Genet..

[34] Matera A.G., Wang Z. (2014). A day in the life of the spliceosome. Nat. Rev. Mol. Cell Biol..

[35] Hansen T.B., Jensen T.I., Clausen B.H., Bramsen J.B., Finsen B., Damgaard C.K., Kjems J. (2013). Natural RNA circles function as efficient microRNA sponges. Nature.

[36] Guo J.U., Agarwal V., Guo H., Bartel D.P. (2014). Expanded identification and characterization of mammalian circular RNAs. Genome Biol..

[37] Ashwal-Fluss R., Meyer M., Pamudurti N.R., Ivanov A., Bartok O., Hanan M., Evantal N., Memczak S., Rajewsky N., Kadener S. (2014). circRNA Biogenesis Competes with Pre-mRNA Splicing. Mol. Cell.

[38] Zhou M.-Y., Yang J.-M., Xiong X.-D. (2018). The emerging landscape of circular RNA in cardiovascular diseases. J. Mol. Cell. Cardiol..

[39] Altesha M., Ni T., Khan A., Liu K., Zheng X. (2019). Circular RNA in cardiovascular disease. J. Cell. Physiol..

[40] Zhang Y., Zhang X.-O., Chen T., Xiang J.-F., Yin Q.-F., Xing Y.-H., Zhu S., Yang L., Chen L.-L. (2013). Circular Intronic Long Noncoding RNAs. Mol. Cell.

[41] Legnini I., Di Timoteo G., Rossi F., Morlando M., Briganti F., Sthandier O., Fatica A., Santini T., Andronache A., Wade M. (2017). Circ-ZNF609 Is a Circular RNA that Can Be Translated and Functions in Myogenesis. Mol. Cell.

[42] Yang Y., Fan X., Mao M., Song X., Wu P., Zhang Y., Jin Y., Yang Y., Chen L.-L., Wang Y. (2017). Extensive translation of circular RNAs driven by N6-methyladenosine. Cell Res..

[43] Pamudurti N.R., Bartok O., Jens M., Ashwal-Fluss R., Stottmeister C., Ruhe L., Hanan M., Wyler E., Perez-Hernandez D., Ramberger E. (2017). Translation of CircRNAs. Mol. Cell.

[44] Yang Y., Gao X., Zhang M., Yan S., Sun C., Xiao F., Huang N., Yang X., Zhao K., Zhou H. (2018). Novel Role of FBXW7 Circular RNA in Repressing Glioma Tumorigenesis. JNCI J. Natl. Cancer Inst..

[45] Fan X., Weng X., Zhao Y., Chen W., Gan T., Xu D. (2017). Circular RNAs in Cardiovascular Disease: An Overview. BioMed Res. Int..

[46] Suzuki H. (2006). Characterization of RNase R-digested cellular RNA source that consists of lariat and circular RNAs from pre-mRNA splicing. Nucleic Acids Res..

[47] Wang W., Wang Y., Piao H., Li B., Huang M., Zhu Z., Li D., Wang T., Xu R., Liu K. (2019). Circular RNAs as potential biomarkers and therapeutics for cardiovascular disease. PeerJ.

[48] Zhu L., Li N., Sun L., Zheng D., Shao G. (2021). Non-coding RNAs: The key detectors and regulators in cardiovascular disease. Genomics.

[49] Wang Z. (2013). miRNAin the Regulation of Ion Channel/Transporter Expression. Compr. Physiol..

[50] Choi E., Cha M.-J., Hwang K.-C. (2014). Roles of Calcium Regulating MicroRNAs in Cardiac Ischemia-Reperfusion Injury. Cells.

[51] Ikeda S., He A., Kong S.W., Lu J., Bejar R., Bodyak N., Lee K.-H., Ma Q., Kang P.M., Golub T.R. (2009). MicroRNA-1 Negatively Regulates Expression of the Hypertrophy-Associated Calmodulin and Mef2a Genes. Mol. Cell. Biol..

[52] Gurha P., Wang T., Larimore A.H., Sassi Y., Abreu-Goodger C., Ramirez M.O., Reddy A.K., Engelhardt S., Taffet G.E., Wehrens X. (2013). microRNA-22 Promotes Heart Failure through Coordinate Suppression of PPAR/ERR-Nuclear Hormone Receptor Transcription. PLoS ONE.

[53] Huang Z., Chen J., Seok H.Y., Zhang Z., Kataoka M., Hu X., Wang D.-Z. (2013). MicroRNA-22 Regulates Cardiac Hypertrophy and Remodeling in Response to Stress. Circ. Res..

[54] Li R.-C., Tao J., Guo Y.-B., Wu H.-D., Liu R.-F., Bai Y., Lv Z.-Z., Luo G.-Z., Li L.-L., Wang M. (2013). In Vivo Suppression of MicroRNA-24 Prevents the Transition Toward Decompensated Hypertrophy in Aortic-Constricted Mice. Circ. Res..

[55] Xu M., Wu H.-D., Li R.-C., Zhang H.-B., Wang M., Tao J., Feng X.-H., Guo Y.-B., Li S.-F., Lai S.-T. (2012). Mir-24 regulates junctophilin-2 expression in cardiomyocytes. Circ. Res..

[56] Dong D.-L., Chen C., Huo R., Wang N., Li Z., Tu Y.-J., Hu J.-T., Chu X., Huang W., Yang B.-F. (2010). Reciprocal Repression Between MicroRNA-133 and Calcineurin Regulates Cardiac Hypertrophy. Hypertension.

[57] van Rooij E., Sutherland L.B., Liu N., Williams A.H., McAnally J., Gerard R.D., Richardson J.A., Olson E.N. (2006). A signature pattern of stress-responsive microRNAs that can evoke cardiac hypertrophy and heart failure. Proc. Natl. Acad. Sci. USA.

[58] Lin Z., Murtaza I., Wang K., Jiao J., Gao J., Li P.-F. (2009). miR-23a functions downstream of NFATc3 to regulate cardiac hypertrophy. Proc. Natl. Acad. Sci. USA.

[59] Callis T.E., Pandya K., Seok H.Y., Tang R.-H., Tatsuguchi M., Huang Z.-P., Chen J.-F., Deng Z., Gunn B., Shumate J. (2009). MicroRNA-208a is a regulator of cardiac hypertrophy and conduction in mice. J. Clin. Investig..

[60] Kim J.O., Song D.W., Kwon E.J., Hong S.-E., Song H.K., Min C.K., Kim D.H. (2015). miR-185 Plays an Anti-Hypertrophic Role in the Heart via Multiple Targets in the Calcium-Signaling Pathways. PLoS ONE.

[61] Heymans S., Corsten M.F., Verhesen W., Carai P., van Leeuwen R.E., Custers K., Peters T., Hazebroek M., Stöger L., Wijnands E. (2013). Macrophage MicroRNA-155 Promotes Cardiac Hypertrophy and Failure. Circulation.

[62] Seok H.Y., Chen J., Kataoka M., Huang Z., Ding J., Yan J., Hu X., Wang D.-Z. (2014). Loss of MicroRNA-155 Protects the Heart From Pathological Cardiac Hypertrophy. Circ. Res..

[63] Wang J., Song Y., Zhang Y., Xiao H., Sun Q., Hou N., Guo S., Wang Y., Fan K., Zhan D. (2011). Cardiomyocyte overexpression of miR-27b induces cardiac hypertrophy and dysfunction in mice. Cell Res..

[64] Yang T., Zhang G.-F., Chen X.-F., Gu H.-H., Fu S.-Z., Xu H.-F., Feng Q., Ni Y.-M. (2013). MicroRNA-214 provokes cardiac hypertrophy via repression of EZH2. Biochem. Biophys. Res. Commun..

[65] Yang Y., Del Re D.P., Nakano N., Sciarretta S., Zhai P., Park J., Sayed D., Shirakabe A., Matsushima S., Park Y. (2015). miR-206 Mediates YAP-Induced Cardiac Hypertrophy and Survival. Circ. Res..

[66] Wang Y.-S., Zhou J., Hong K., Cheng X.-S., Li Y.-G. (2015). MicroRNA-223 Displays a Protective Role Against Cardiomyocyte Hypertrophy by Targeting Cardiac Troponin I-Interacting Kinase. Cell. Physiol. Biochem..

[67] Bao J., Lu Y., She Q.-Y., Dou W., Tang R., Xu X., Zhang M., Zhu L., Zhou Q., Li H. (2021). MicroRNA-30 regulates left ventricular hypertrophy in chronic kidney disease. JCI Insight.

[68] Cheng Y., Zhang C. (2010). MicroRNA-21 in Cardiovascular Disease. J. Cardiovasc. Transl. Res..

[69] Kura B., Kalocayova B., Devaux Y., Bartekova M. (2020). Potential Clinical Implications of miR-1 and miR-21 in Heart Disease and Cardioprotection. Int. J. Mol. Sci..

[70] Cheng Y., Ji R., Yue J., Yang J., Liu X., Chen H., Dean D.B., Zhang C. (2007). MicroRNAs Are Aberrantly Expressed in Hypertrophic Heart: Do They Play a Role in Cardiac Hypertrophy?. Am. J. Pathol..

[71] Sayed D., Hong C., Chen I.-Y., Lypowy J., Abdellatif M. (2007). MicroRNAs Play an Essential Role in the Development of Cardiac Hypertrophy. Circ. Res..

[72] Thum T., Gross C., Fiedler J., Fischer T., Kissler S., Bussen M., Galuppo P., Just S., Rottbauer W., Frantz S. (2008). MicroRNA-21 contributes to myocardial disease by stimulating MAP kinase signalling in fibroblasts. Nature.

[73] Sayed D., Rane S., Lypowy J., He M., Chen I.-Y., Vashistha H., Yan L., Malhotra A., Vatner D., Abdellatif M. (2008). MicroRNA-21 Targets Sprouty2 and Promotes Cellular Outgrowths. Mol. Biol. Cell.

[74] Jiang C., Ding N., Li J., Jin X., Li L., Pan T., Huo C., Li Y., Xu J., Li X. (2018). Landscape of the long non-coding RNA transcriptome in human heart. Briefings Bioinform..

[75] Liu C.-F., Tang W.W. (2019). Epigenetics in Cardiac Hypertrophy and Heart Failure. JACC Basic Transl. Sci..

[76] Chouvarine P., Photiadis J., Cesnjevar R., Scheewe J., Bauer U.M., Pickardt T., Kramer H.-H., Dittrich S., Berger F., Hansmann G. (2021). RNA expression profiles and regulatory networks in human right ventricular hypertrophy due to high pressure load. iScience.

[77] Yan Y., Tang R., Li B., Cheng L., Ye S., Yang T., Han Y.-C., Liu C., Dong Y., Qu L.-H. (2021). The cardiac translational landscape reveals that micropeptides are new players involved in cardiomyocyte hypertrophy. Mol. Ther..

[78] Anderson D.M., Anderson K.M., Chang C.-L., Makarewich C.A., Nelson B.R., McAnally J.R., Kasaragod P., Shelton J.M., Liou J., Bassel-Duby R. (2015). A Micropeptide Encoded by a Putative Long Noncoding RNA Regulates Muscle Performance. Cell.

[79] Lv L., Li T., Li X., Xu C., Liu Q., Jiang H., Li Y., Liu Y., Yan H., Huang Q. (2018). The lncRNA Plscr4 Controls Cardiac Hypertrophy by Regulating miR-214. Mol. Ther. Nucleic Acids.

[80] Wang K., Liu F., Zhou L.-Y., Long B., Yuan S.-M., Wang Y., Liu C.-Y., Sun T., Zhang X.-J., Li P.-F. (2014). The Long Noncoding RNA CHRF Regulates Cardiac Hypertrophy by Targeting miR-489. Circ. Res..

[81] Liu L., An X., Li Z., Song Y., Li L., Zuo S., Liu N., Yang G., Wang H., Cheng X. (2016). The H19 long noncoding RNA is a novel negative regulator of cardiomyocyte hypertrophy. Cardiovasc. Res..

[82] Keniry A., Oxley D., Monnier P., Kyba M., Dandolo L., Smits G., Reik W. (2012). The H19 lincRNA is a developmental reservoir of miR-675 that suppresses growth and Igf1r. Nat. Cell Biol..

[83] Jiang F., Zhou X., Huang J. (2016). Long Non-Coding RNA-ROR Mediates the Reprogramming in Cardiac Hypertrophy. PLoS ONE.

[84] Wen Z.-Q., Li S.-H., Shui X., Tang L.-L., Zheng J.-R., Chen L. (2019). LncRNA PEG10 aggravates cardiac hypertrophy through regulating HOXA9. Eur. Rev. Med. Pharmacol. Sci..

[85] Zhou G., Li C., Feng J., Zhang J., Fang Y. (2018). lncRNA UCA1 Is a Novel Regulator in Cardiomyocyte Hypertrophy through Targeting the miR-184/HOXA9 Axis. Cardiorenal. Med..

[86] Yan Y., Zhang B., Liu N., Qi C., Xiao Y., Tian X., Li T., Liu B. (2016). Circulating Long Noncoding RNA UCA1 as a Novel Biomarker of Acute Myocardial Infarction. BioMed. Res. Int..

[87] Yuan Y., Wang J., Chen Q., Wu Q., Deng W., Zhou H., Shen D. (2019). Long non-coding RNA cytoskeleton regulator RNA (CYTOR) modulates pathological cardiac hypertrophy through miR-155-mediated IKKi signaling. Biochim. Biophys. Acta (BBA) Mol. Basis Dis..

[88] Han P., Li W., Lin C.-H., Yang J., Shang C., Nuernberg S.T., Jin K.K., Xu W., Lin C.-Y., Lin C.-J. (2014). A long noncoding RNA protects the heart from pathological hypertrophy. Nature.

[89] Wang Z., Zhang X.-J., Ji Y.-X., Zhang P., Deng K.-Q., Gong J., Ren S., Wang X., Chen I., Wang H. (2016). The long noncoding RNA Chaer defines an epigenetic checkpoint in cardiac hypertrophy. Nat. Med..

[90] Viereck J., Kumarswamy R., Foinquinos A., Xiao K., Avramopoulos P., Kunz M., Dittrich M., Maetzig T., Zimmer K., Remke J. (2016). Long noncoding RNA Chast promotes cardiac remodeling. Sci. Transl. Med..

[91] Lin H., Zhu Y., Zheng C., Hu D., Ma S., Chen L., Wang Q., Chen Z., Xie J., Yan Y. (2021). Antihypertrophic Memory After Regression of Exercise-Induced Physiological Myocardial Hypertrophy Is Mediated by the Long Noncoding RNA Mhrt779. Circulation.

[92] Jakobi T., Czaja-Hasse L.F., Reinhardt R., Dieterich C. (2016). Profiling and Validation of the Circular RNA Repertoire in Adult Murine Hearts. Genom. Proteom. Bioinform..

[93] Werfel S., Nothjunge S., Schwarzmayr T., Strom T.-M., Meitinger T., Engelhardt S. (2016). Characterization of circular RNAs in human, mouse and rat hearts. J. Mol. Cell. Cardiol..

[94] Meng Z., Chen C., Cao H., Wang J., Shen E. (2019). Whole transcriptome sequencing reveals biologically significant RNA markers and related regulating biological pathways in cardiomyocyte hypertrophy induced by high glucose. J. Cell. Biochem..

[95] Sun Y., Jiang X., Lv Y., Liang X., Zhao B., Bian W., Zhang D., Jiang J., Zhang C. (2020). Circular RNA Expression Profiles in Plasma from Patients with Heart Failure Related to Platelet Activity. Biomolecules.

[96] Yang M.-H., Wang H., Han S.-N., Jia X., Zhang S., Dai F.-F., Zhou M.-J., Yin Z., Wang T.-Q., Zang M.-X. (2020). Circular RNA expression in isoproterenol hydrochloride-induced cardiac hypertrophy. Aging.

[97] Liu N., Bezprozvannaya S., Williams A.H., Qi X., Richardson J.A., Bassel-Duby R., Olson E.N. (2008). microRNA-133a regulates cardiomyocyte proliferation and suppresses smooth muscle gene expression in the heart. Genes Dev..

[98] Carè A., Catalucci D., Felicetti F., Bonci D., Addario A., Gallo P., Bang M.-L., Segnalini P., Gu Y., Dalton N.D. (2007). MicroRNA-133 controls cardiac hypertrophy. Nat. Med..

[99] Lim T.B., Aliwarga E., Luu T.D.A., Li Y.P., Ng S.L., Annadoray L., Sian S., Ackers-Johnson M.A., Foo R.S.-Y. (2019). Targeting the highly abundant circular RNA circSlc8a1 in cardiomyocytes attenuates pressure overload induced hypertrophy. Cardiovasc. Res..

[100] Li M., Ding W., Tariq M.A., Chang W., Zhang X., Xu W., Hou L., Wang Y., Wang J. (2018). A circular transcript of ncx1 gene mediates ischemic myocardial injury by targeting miR-133a-3p. Theranostics.

[101] Deng Y., Wang J., Xie G., Zeng X., Li H. (2019). Circ-HIPK3 Strengthens the Effects of Adrenaline in Heart Failure by MiR-17-3p—ADCY6 Axis. Int. J. Biol. Sci..

[102] Xu X., Wang J., Wang X. (2020). Silencing of circHIPK3 Inhibits Pressure Overload-Induced Cardiac Hypertrophy and Dysfunction by Sponging miR-185-3p. Drug Des. Dev. Ther..

[103] Wang W., Wang L., Yang M., Wu C., Lan R., Wang W., Li Y. (2021). Circ-SIRT1 inhibits cardiac hypertrophy via activating SIRT1 to promote autophagy. Cell Death Dis..

[104] Wang K., Long B., Liu F., Wang J., Liu C.-Y., Zhao B., Zhou L.-Y., Sun T., Wang M., Cui-Yun L. (2016). A circular RNA protects the heart from pathological hypertrophy and heart failure by targeting miR-223. Eur. Heart J..

[105] Li H., Xu J.-D., Fang X.-H., Zhu J.-N., Yang J., Pan R., Yuan S.-J., Zeng N., Yang Z.-Z., Yang H. (2019). Circular RNA circRNA_000203 aggravates cardiac hypertrophy via suppressing miR-26b-5p and miR-140-3p binding to Gata4. Cardiovasc. Res..

[106] Satoh T., Wang L., Espinosa-Diez C., Wang B., Hahn S.A., Noda K., Rochon E.R., Dent M.R., Levine A.R., Baust J.J. (2021). Metabolic Syndrome Mediates ROS-miR-193b-NFYA–Dependent Downregulation of Soluble Guanylate Cyclase and Contributes to Exercise-Induced Pulmonary Hypertension in Heart Failure With Preserved Ejection Fraction. Circulation.

[107] Sato M., Kadomatsu T., Miyata K., Warren J.S., Tian Z., Zhu S., Horiguchi H., Makaju A., Bakhtina A., Morinaga J. (2021). The lncRNA Caren antagonizes heart failure by inactivating DNA damage response and activating mitochondrial biogenesis. Nat. Commun..

[108] Zhang Y., Chen B. (2021). Silencing circ_0062389 alleviates cardiomyocyte apoptosis in heart failure rats via modulating TGF-β1/Smad3 signaling pathway. Gene.

[109] Täubel J., Hauke W., Rump S., Viereck J., Batkai S., Poetzsch J., Rode L., Weigt H., Genschel C., Lorch U. (2021). Novel antisense therapy targeting microRNA-132 in patients with heart failure: Results of a first-in-human Phase 1b randomized, double-blind, placebo-controlled study. Eur. Heart J..

[110] Crooke S.T., Witztum J.L., Bennett C.F., Baker B.F. (2019). RNA-Targeted Therapeutics. Cell Metab..

[111] De Majo F., De Windt L.J. (2018). RNA therapeutics for heart disease. Biochem. Pharmacol..

[112] Huang J., Chen M., Chen D., Gao X.-C., Zhu S., Huang H., Hu M., Zhu H., Yan G.-R. (2017). A Peptide Encoded by a Putative lncRNA HOXB-AS3 Suppresses Colon Cancer Growth. Mol. Cell.

[113] Zheng X., Chen L., Zhou Y., Wang Q., Zheng Z., Xu B., Wu C., Zhou Q., Hu W., Wu C. (2019). A novel protein encoded by a circular RNA circPPP1R12A promotes tumor pathogenesis and metastasis of colon cancer via Hippo-YAP signaling. Mol. Cancer.

[114] Kong S., Tao M., Shen X., Ju S. (2020). Translatable circRNAs and lncRNAs: Driving mechanisms and functions of their translation products. Cancer Lett..

